# Low-level soluble chloride extraction in soil

**DOI:** 10.1016/j.mex.2020.100967

**Published:** 2020-06-22

**Authors:** Daniel Larsen, Brian Waldron

**Affiliations:** Department of Earth Sciences, Center for Applied Earth Science and Engineering Research, University of Memphis, Memphis, TN 38152, USA

**Keywords:** Recharge, Rinses, Fluid:soil ratio

## Abstract

Three methods of extraction of low-level soluble chloride contents from vadose-zone soil were evaluated in this study. Three methods were employed on a silty sand soil using a 2:1 fluid:soil ratio: 1) Method A utilized three successive rinses with deionized water; 2) Method B applied three successive rinses of 0.0001 M and 0.001 M Na_2_SO_4_ solution; and 3) Method C passed deionized water through the soil with a pressurized filtration system three times. Method A had lower standard deviation and yielded more consistent soluble chloride contents per rinse than method C; Method B was ruled out because of concerns that the Na_2_SO_4_ reagent contained trace amounts of chloride. Method A was applied with a 1:1 fluid:soil ratio in duplicate to 50 samples from a 34-m thick vadose-zone borehole, yielding a mean difference in duplicates of 13.9% and percent total extracted soluble chloride of 62.4 ± 9.9%, 25.2 ± 7.4%, and 12.4 ± 6.6% in each of the three successive rinses.

• Three successive rinses of soil with deionized water achieved consistent extraction results.
• Multiple rinses are necessary to extract soluble chloride if chloride contents are low.
• This method is amenable to analysis of soil in vadose-zone borehole samples.

## Graphical abstract

**Figure:**
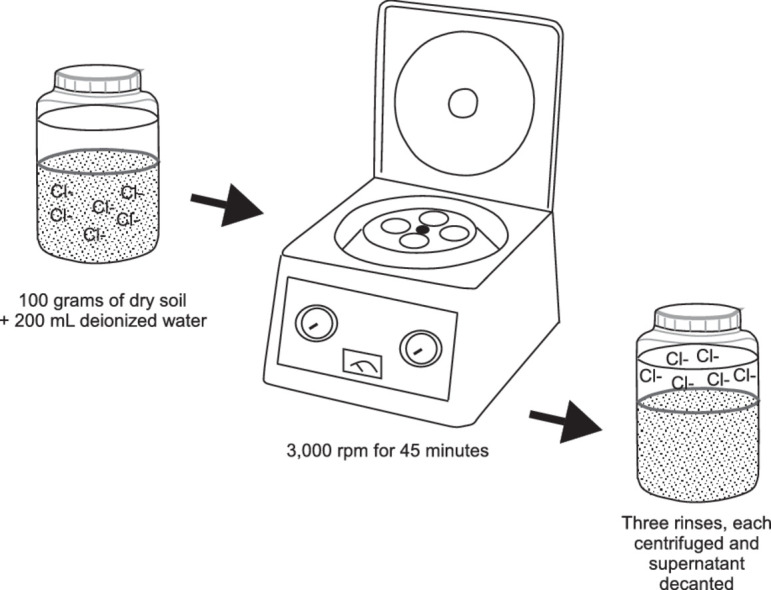

Specifications table

**Table:** 

Subject Area	Earth and Planetary Sciences
More specific subject area	*Soil chemical extraction*
Method name	*Low-level soluble chloride extraction*
Name and reference of original method	[1]*. The use of environmental chloride and tritium to estimate total recharge to an unconfined aquifer, Australian Journal of Soil Research, v. 16, pp. 181–195.*
Resource availability	*If applicable, include links to resources necessary to reproduce the method (e.g. data, software, hardware, reagent)*

## Introduction

Chloride mass balance (CMB) is a method for estimating recharge in semi-arid to humid regions [1,^5^,^9^,[10], [11]. Determining low-level concentrations of extractable chloride in soils is essential for application of the CMB in vadose-zone studies. A critical source of error in the recharge estimate is the chloride accounting in the soil and precipitation; thus, improved total chloride extraction and precision are essential to obtain better estimates of recharge.

A common limitation of the CMB method in humid settings is the limit of detection for chloride from soil extractions [10]. Most previous studies have used a chloride extraction method consisting of mixing soil and deionized water in a consistent ratio and then separating the fluid from the soil by centrifuge followed by filtration. Fluid:soil ratios applied range from ~1:1 [5,^8^,12] to 600:1 [4], depending on soluble chloride concentrations in the soil. Studies by Murphy et al. [7] showed chloride extraction was sensitive to the fluid:soil ratio, especially for silt loam soils. Typical methods of chloride analysis include ion chromatography [5], [8], [12] and colorimetry using mercuric thiocyanate [2,^3^,^6^,^13^,14]. It is unclear whether many previous studies have optimized the chloride extraction method or attempted other methods of chloride removal; thus, three methods of extraction were evaluated to maximize chloride extraction and reproducibility. Extraction fluids included deionized water, which has been commonly applied in other studies [1,^5^,^8^,12], and Na_2_SO_4_ solutions to evaluate whether exchangeable chloride on soil surfaces contributes to the soluble chloride content. Extraction methods included rinsing soil with extraction fluids followed by centrifuge separation and filtration of extraction fluids. The method of extraction yielding the most consistent and reproducible results was used to determine low-level chloride contents in samples from a 34-m vadose-zone borehole in western Tennessee, U.S.A.

## Materials

Soil samples for evaluating extraction methods were obtained from push-core and auger-flight samples at 0.3 m intervals in an 8-m borehole through loess (windblown silt) and sand on an upland surface in southeastern Fayette County, Tennessee (longitude −89.197°, latitude 35.031°). The 34-m vadose-zone borehole samples were obtained from hollow-stem auger tubes at a borehole at Pinecrest Presbyterian Retreat (longitude −89.275°, latitude 35.053°). The hollow-stem auger tubes were 152-cm long and fitted with two 76-cm long bicarbonate sleeves. Soil samples were captured within the bicarbonate sleeves and were tightly sealed upon extraction with aluminum foil, a plastic cap and tape. Many of the bicarbonate sleeve pairs arrived at the surface partially filled, which is likely a result of compaction due to friction between the soil and the sleeve surface.

The reagents and supplies used for the extraction analysis were determined to have minimal chloride concentrations, either from assay data on the reagents or by verification from the manufacturer. Neither of the fields from which the soil materials were obtained had been treated with chlorine-bearing pesticides, herbicides, or fertilizers.

## Method

Soil samples (~1 kg each) from the 8-m borehole were homogenized in an industrial mixer to yield a consistent material comprising 70% sand, and 30% silt and clay. Units and definitions are presented in the Appendix. The homogenized soil was weighed into 100-g subsamples for extraction analysis. Three chloride extraction methods were investigated to determine their relative effectiveness and consistency in removing soluble chloride from the homogenized soil sample (Table 1). Each method was applied to 4 subsamples of the homogenized soil. Blank analyses were performed for each method using the same procedure but without soil.

**Table 1:** Comparative results from soluble chloride extraction of silty sand soil using methods A, B, and C; Extracted Cl^−^ is ppm in dry soil.

Experiments used approximately 100 gr. of sandy soil and 200 mL of deionzied water or NaSO4 solution					
	Rinse 1	Rinse 2	Rinse 3	Total	Extracted
**Method A**	Cl^−^(mg/L)	Cl^−^(mg/L)	Cl^−^(mg/L)	Cl^−^(mg/L)	Cl^−^(ppm)
A1	0.51	0.28	0.11	0.90	
A2	0.76	0.32	0.17	1.25	
A3	0.78	0.26	0.21	1.25	
A4	0.76	0.34	0.2	1.30	
Mean	0.70	0.30	0.17	1.18	
Standard Deviation	0.13	0.04	0.05	0.18	
Experimental Blank	C	0.19	0.20		
Mean - Blank	0.51	0.11	−0.03	0.62	3.71
% total Cl^−^	82.19	17.81	0.00	100.00	
					
**Method BL: 0.0001** **M Na_2_SO_4_**					
BL1	0.83	0.26	0.18	1.27	
BL2	0.84	0.29	0.31	1.44	
BL3	0.80	0.28	0.24	1.32	
BL4	0.80	0.27	0.18	1.25	
Mean	0.82	0.28	0.23	1.32	
Standard Deviation	0.02	0.01	0.06	0.09	
Experimental Blank	0.17	0.18	0.18		
Mean - Blank	0.65	0.10	0.05	0.79	4.74
% total Cl^−^	81.96	12.03	6.01	100.00	
					
**Method BH: 0.001** **M Na_2_SO_4_**					
BH1	1.51	0.40	0.19	2.10	
BH2	1.43	0.42	0.22	2.07	
BH3	1.43	0.44	0.25	2.12	
BH4	1.46	0.54	0.3	2.30	
Mean	1.46	0.45	0.24	2.15	
Standard Deviation	0.04	0.06	0.05	0.10	
Experimental Blank	0.38	0.13	0.18		
Mean - Blank	1.08	0.32	0.06	1.46	8.75
% total Cl^−^	73.93	21.96	4.12	100.00	
					
**Method C: Column**					
C1	0.61	0.07	0.08	0.76	
C2	1.10	0.42	0.04	1.56	
C3	1.00	0.09	BD	1.09	
C4	0.90	0.1	0.33	1.33	
Mean	0.90	0.17	0.15	1.19	
Standard Deviation	0.21	0.17	0.16	0.34	
Experimental Blank	0.15	0.19	0.16		
Mean - Blank	0.75	−0.02	−0.01	0.75	4.52
% total Cl^−^	100.00	0.00	0.00	100.00	

C: Contaminated.

BD: below detection.

Methods A and B used a 2:1 fluid:soil ratio with 100 g homogenized soil samples and 200 mL of extraction fluid. In methods A and B, the soil-fluid solution was hand-stirred for five minutes, mechanically shaken for approximately 12 h, and centrifuged at 3000 rpm for 45 min. Method A used deionized water with a resistance of >17.5 Mohm-cm as the extraction fluid. Method B used solutions with concentrations of 0.0001 and 0.001 M Na_2_SO_4_. For both methods A and B, the supernatant from the centrifuge was extracted and passed through a Whatman 40 ashless filter (filter contains < 80 µg/g chlorine) to remove fine particulate matter. The extraction process was repeated two additional times on the same soil sample for a total of three extraction rinses.

Method C used a Nalgene 250 ml filter column with an Osmonics cellulosic filter (0.22 µm). Soil (100 g) and deionized water at a 1:1 ratio were placed in the column, the column was sealed, and nitrogen gas was applied to the top of the column at 260 mm Hg to push the water through the soil sample. The filtered water was collected and reapplied to the top of the column for a second pass, creating an equivalent 2:1 fluid:soil ratio. The extraction procedure was repeated two additional times on the same soil sample to obtain three extraction rinses.

The rinse solutions from each extraction were analyzed using a Dionex DX-120 ion chromatography unit (IC). The IC utilized AG-14 and AS-14 columns with electrolytic suppression and a 25 µL sampling loop for comparative analysis of methods A, B and C. The eluent was a 2.1 mM Na_2_CO - 30.8 mM NaHCO_3_ buffer solution prepared from high-purity standards. Reproducibility of 0.2 and 2.0 mg/L Cl^−^ standards were ±15% and ±1.8%, respectively.

Method A was applied with a 1:1 fluid:soil ratio in duplicate to samples from the 34-m vadose-zone borehole. The borehole samples were homogenized from half-core splits of 10-cm diameter core and ranged from 50 to 100 g in mass, based on available soil material. The IC conditions were the same as above except that the sample loop volume was increased to 200 µL to improve the reproducible detection limit. The blank-subtracted chloride contents from the three rinses were summed and divided by the gravimetric water content in 100 g of sample to determine the chloride concentration in the vadose zone water.

For the vadose-zone borehole samples, gravimetric moisture contents were determined by weighing 400 g of homogenized sample, drying the sample at 105 °C for 12 h, weighing the dried soil, and determining the water content by weight loss.

## Extraction and analytical results

The chloride concentrations in rinses 1 through 3 in four replicates for methods A, B and C are tabulated in Table 1, with the BL series representing the 0.0001 M Na_2_SO_4_ solution and BH series representing the 0.001 M Na_2_SO_4_ solution. Analytical blank results are provided for each method and subtracted from the mean chloride concentrations of the replicates to calculate the total soluble extracted chloride content as ppm in dry soil. The experimental blank for Method A, rinse 1 was contaminated; the average of the experimental blank values for rinses 2 and 3 was subtracted from rinse 1. For rinses in which the experimental blank was greater than the mean extracted chloride, a negative extracted chloride content was calculated and no chloride was added to the total.

Methods A, BL, BH, and C yielded dissimilar total soluble extracted chloride of 3.71, 4.74, 8.75, and 4.52 ppm, respectively. The standard deviations for replicates of methods A, BL, and BH were one-half to one-third of the deviations in method C. Chloride concentrations in rinses from individual replicates of method C are highly erratic, sometimes varying by close to an order of magnitude. The blank concentrations for individual rinses of methods A, BL, and C were similar and lower than the mean blank concentration of method BH. The higher blank concentration of method BH suggests that some chloride may be in the Na_2_SO_4_ reagent. This may explain the higher extracted chloride content for method BH.

Given the erratic soluble extracted chloride concentrations from method C and the potential for trace quantities of chloride in the Na_2_SO_4_ reagent in methods BL and BH, method A was chosen for soluble chloride extraction in the vadose-zone borehole samples. Because of the low total soluble chloride content of the homogenized soil (Table 1), the results from method A indicate a lower fluid:soil ratio may be beneficial for achieving chloride concentrations above the blank concentration in all rinses. In regard to IC analysis, the sample loop volume was increased from 25 to 200 µl to improve instrument sensitivity.

The soluble chloride extraction results for the vadose-zone borehole samples are tabulated in Table 2 as ppm in dry soil. The mean replicate difference was 13.9% with a standard deviation of 15.6%. The relatively high standard deviation may reflect uneven distribution of chloride in the sample. For the total soluble chloride extracted, 62.4 ± 9.8% (1 standard deviation) was removed during the first rinse, whereas 25.2 ± 7.4% and 12.4 ± 6.6% were extracted during the second and third rinses, respectively. The extracted soluble chloride results indicate the need for multiple rinses because only ~2/3 of the soluble chloride was removed during the first rinse. The mean chloride concentration in the analytical blanks was 0.04 ± 0.02 mg/L and that of the experimental blanks was 0.04 ± 0.01 mg/L. The blank results indicate that little chloride was added during the experimental procedure. The increased sample loop volume decreased the experimental chloride blank concentrations from 0.2 mg/L (data in Table 1) to 0.04 mg/L (data in Table 2), which improved the detection limit of the IC analysis.

**Table 2:** Soluble chloride extraction results 34-m vadose-zone borehole using modified method A.

	Rinse A1	Rinse A2	%	Extracted Cl^−^	%	%	%
Sample	(ppm)	(ppm)	Difference	(ppm)	First rinse	Second rinse	Third rinse
C.1/2.1-A-R1	1.523	1.390	9.1	1.456	53.9	35.6	10.5
C.2.2-A-R1	0.446	0.579	26.0	0.512	51.2	38.6	10.2
C.3.2-B-R1	0.992	0.820	19.1	0.906	48.9	25.7	25.4
C.4.1-A-R2	0.584	0.314	60.3	0.449	57.5	37.6	4.9
C.4.2-B-R2	0.686	0.601	13.2	0.643	54.4	39.5	6.2
C.5.1-A-R2	0.759	0.751	1.0	0.755	68.8	22.5	8.6
C.5.2-B-R2	0.899	0.910	1.2	0.904	61.2	31.1	7.8
C.6.1-B-R1	1.140	1.390	19.8	1.265	65.5	27.8	6.6
C.6.2-A-R1	1.080	1.252	14.7	1.166	62.6	24.7	12.7
C.7/8.1-A-R1	0.833	1.051	23.2	0.942	56.3	29.4	14.3
C.8.2-B-R1	1.043	0.942	10.2	0.992	66.0	22.8	11.2
C.9/10.1-A-R1	0.595	0.570	4.4	0.583	70.0	22.2	7.8
C.10.2-B-R1	0.543	0.577	6.0	0.560	64.4	27.3	8.3
C.11.1-B-R2	0.756	1.029	30.6	0.893	58.6	22.1	19.3
C.12/13.1-B-R2	0.646	0.690	6.6	0.668	56.4	29.4	14.2
C.13.1-A-R2	0.484	0.482	0.5	0.483	57.3	26.1	16.6
C.14.1-B-R2	0.437	0.374	15.6	0.406	57.5	24.0	18.5
C.14.2-A-R2	0.552	0.574	4.0	0.563	61.4	22.5	16.1
C.15/16.1-B-R2	0.448	0.698	43.6	0.573	48.4	39.2	12.4
C.16.2-A-R2	0.342	0.375	9.1	0.358	54.1	26.2	19.7
C.17/18.1-B-R2	0.662	0.805	19.4	0.734	58.5	26.5	14.9
C.18.2-A-R2	0.616	0.672	8.8	0.644	57.3	24.2	18.4
C.19/20.1-B-R2	0.387	0.416	7.3	0.402	60.3	24.2	15.4
C.20.2-A-R2	0.235	0.242	3.0	0.239	72.1	22.2	5.7
C.21/22.1-B-R2	0.274	0.242	12.4	0.258	69.4	20.6	9.9
C.22.2-A-R2	0.127	0.150	16.5	0.138	60.4	24.5	15.1
C.23/24.1-B-R2	0.349	0.846	83.1	0.598	38.4	54.6	7.0
C.24.2-A-R2	0.186	0.285	42.1	0.236	82.8	13.6	3.5
C.25/26.1-B-R2	0.192	0.249	26.1	0.220	53.4	30.6	16.0
C.26.2-A-R2	0.312	0.338	7.9	0.325	54.9	32.6	12.4
C.27/28.1-B-R2	0.517	0.500	3.3	0.509	60.8	24.6	14.5
C.28.2-A-R2	0.200	0.185	7.8	0.193	75.2	20.2	4.6
C.29/30.1-B-R2	0.197	0.224	12.5	0.211	72.2	19.7	8.1
C.30.2-A-R2	0.244	0.313	25.0	0.279	62.6	25.7	11.7
C.31/32.1-B-R2	0.287	0.335	15.2	0.311	66.2	20.3	13.4
C.32.2-A-R2	0.209	0.213	2.0	0.211	72.2	17.9	9.9
C.33/34.1-B-R2	0.249	0.256	2.8	0.252	52.5	16.8	30.7
C.34.2-A-R2	0.234	0.284	19.4	0.259	60.0	29.4	10.6
C.35/36.1-B-R2	0.382	0.462	18.8	0.422	71.5	13.1	15.4
C.36.2-A-R2	0.615	0.687	11.0	0.651	81.1	14.5	4.4
C.37/38.1-B-R2	0.543	0.513	5.8	0.528	72.8	23.1	4.1
C.38.2-A-R2	1.241	1.269	2.2	1.255	75.6	19.7	4.7
C.39/40.1-B-R2	0.755	0.768	1.7	0.761	76.9	18.4	4.6
C.40.2-A-R2	0.536	0.543	1.2	0.540	70.0	20.7	9.2
C.41/42.1-B-R2	0.507	0.448	12.3	0.477	67.2	27.0	5.8
C.42.2-A-R2	0.432	0.461	6.6	0.447	63.4	27.0	9.6
C.43/44.1-B-R2	0.504	0.407	21.4	0.455	60.8	27.4	11.8
C.44.2-A-R2	0.403	0.410	1.6	0.406	47.4	27.6	25.0
C.45/46.1-B-R2	0.331	0.295	11.5	0.313	38.9	30.5	30.6
C.46.2-A-R2	0.379	0.502	27.8	0.440	50.3	26.9	22.7
Mean	0.508	0.556	13.9	ND	62.4	25.2	12.4
Standard Deviation	0.305	0.319	15.6	ND	9.8	7.4	6.6

ND: not determined.

## Conclusions

The results of soil extraction of soluble chloride using 2:1 fluid:soil ratio and three rinses of: A) deionized water, B) 0.0001 M and 0.001 M Na_2_SO_4_ solutions, C) deionized water in a pressurized filtration systems support the use of deionized water rinses and centrifuge separation for soluble chloride extraction. The Na_2_SO_4_ solutions may have trace quantities of chloride and the pressurized filtration system yielded variable extraction results. Three rinses are needed to obtain more complete extraction of the total soluble chloride, especially in soils with low soluble chloride contents.

Application of modified method A (1:1 fluid:soil ratio, replicate analysis) to samples from a 34-m vadose-zone borehole yielded reproducibility within 13.9% and consistent percentages of extracted chloride in the three rinses (62.4%, 25.2%, and 12.4%, respectively). Analytical and experimental blanks had similar values, both of which were generally less than the soluble chloride extracted in the third rinse. The results suggest that method A may be useful in other settings where the vadose-zone CMB method is applied to samples containing substantial fine-grained fractions and low soluble chloride contents.

## Declaration of Competing Interest

The authors declare that they have no known competing financial interests or personal relationships that could have appeared to influence the work reported in this paper.

## Figures and Tables

**Table:** 

Unit abbreviation	Unit name	Definition

g, kg	gram, kilogram	mass of solid material
L, mL, µL	liter, milliliter, microliter	volume of fluid
m , cm, µm	meter, centimeter, micrometer	measure of distance
°C	degrees Centigrade	measure of temperature
Mohm-cm	Mega-ohm per centimeter	measure of fluid electrical resistance
mm Hg	millimeters of mercury	air pressure
rpm	revolutions per minute	frequency of rotation
M, mM	molarity, millimolarity	molar concentration of solute per liter of water
µg/g	microgram per gram	gram concentration of chemical per gram of solid material
mg/L	milligrams per liter	mass concentration per fluid volume
ppm	grams per million grams	gram concentration of chemical per million grams of solid material
